# Modified quadruple inversion recovery prepulse for arterial spin labeling angiography without the need of subtraction

**DOI:** 10.1186/1532-429X-13-S1-P375

**Published:** 2011-02-02

**Authors:** Marcelo E Andia, Rene M Botnar

**Affiliations:** 1 Division of Imaging Sciences, King’s College London, St Thomas’ Hospital, London, UK

## Introduction

**Figure 1:**
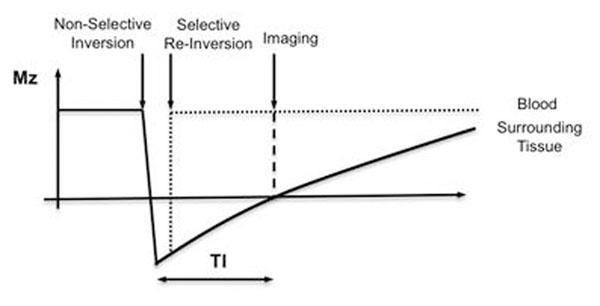
Double inversion bright-blood MR angiography sequence

Arterial Spin Labeling (ASL) is a well-known technique that allows the non-invasive acquisition of angiograms without the need of a contrast agent [1][2]. ASL angiography is still clinically underused because of several challenges [3]: ASL requires two acquisitions (labeled and non labeled dataset) thereby doubling scan time. The need of subtraction increases the sensitivity to spatial misregistration and the need of the choice of an optimal inversion delay for best blood-to-background contrast introduces some “operator dependence”. An alternative technique not requiring image subtraction has been proposed (Figure 1) [4]. This approach is based on a double inversion (DIR-ASL) prepulse and provides good background suppression if the surrounding tissues have similar T1 values (Figure 1). However, DIR-ASL has only one optimal inversion delay and thus there is a trade-off between background suppression and visualized vessel extent. Our goal was to develop an ASL technique with improved background suppression and without the need of subtraction.

## Methods

**Figure 2:**
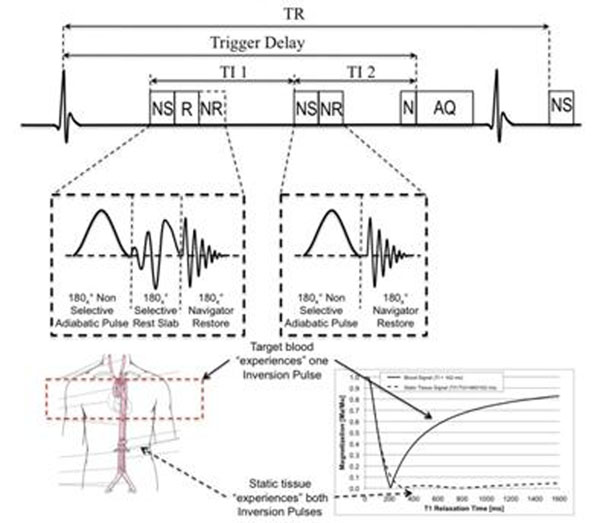
mQIR-ASL sequence, NS: Adiabatic Non-Selective Inversion Pulse. R: Selective Inversion Pulse, NR: Navigator Restore Pulse, N: Naviagtor, AQ: Acquisition

The proposed method is a modified Quadruple Inversion Recovery [5] sequence (mQIR-ASL), which provides excellent background signal suppression according to our simulations (Figure 2). The first pair of RF pulses consists of a non-selective inversion RF pulse followed by a selective reinversion pulse and a navigator-restore pulse. The second pair of RF pulses consists of a non-selective inversion pulse followed by a navigator-restore pulse. With this configuration, the upstream labeled blood only “experiences” the second non-selective inversion pulse, while the static tissue “experiences” both non-selective inversion pulses. With the right choice of TI1 and TI2, signal from static tissue can be suppressed over a wide T1 range while maintaining the signal of target blood (Figure 2). This pre-pulse was implemented on a 3T Achieva Gyroscan MR scanner (Philips Healthcare, Best, NL) and tested in 5 subjects.

## Results

**Figure 3:**
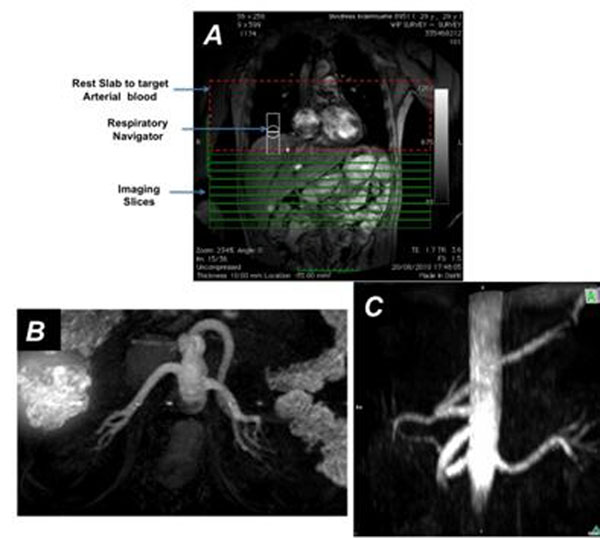
In vivo result with mQIR-ASL (TR/TI1/TI2 = 1000/480/162 ms). A- Planning procedure; B- and C- MIPs of aorta and renal arteries

**Figure 4:**
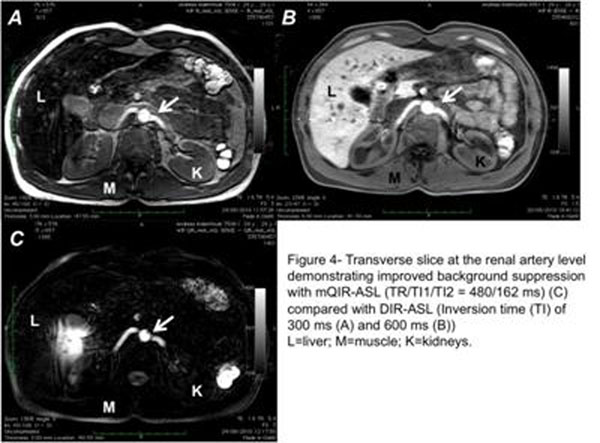
Transverse slice at the renal artery level demonstrating improved background suppression with mQIR-ASL (TW/TI1/TI2 = 480/162 ms) © compared with DIR-ASL (Inversion time (TI) of 300 ms (A) and 600 ms (B)). L=liver; M=muscle; K=kidneys.

Renal arteries including small branches were successfully visualized in all subjects with excellent suppression of background (Figure 3,4). The plan scan and the maximum projection angiogram (MIP) are shown in Figure 3. Improved background suppression was observed with mQIR-ASL compared to DIR-ASL (Figure 4).

## Conclusions

We demonstrate a new ASL approach for non-contrast enhanced MR angiography with excellent background tissue suppression and without the need of subtraction. In comparison to DIR-ASL, mQIR-ASL yielded better background suppression and improved vessel delineation.
